# Interaction of dental pulp stem cells with Biodentine and MTA after exposure to different environments

**DOI:** 10.1590/1678-775720160099

**Published:** 2016

**Authors:** Anastasia Agrafioti, Vasiliki Taraslia, Vanessa Chrepa, Stefania Lymperi, Panos Panopoulos, Ema Anastasiadou, Evangelos G. Kontakiotis

**Affiliations:** 1 National and Kapodistrian University of Athens, School of Dentistry, Department of Endodontics, Athens, Greece.; 2 Biomedical Research Foundation of the Academy of Athens, Department of Genetics and Gene Therapy, Athens, Greece.; 3 University of Washington, Department of Endodontics, Seattle, WA, USA.

**Keywords:** Acidic environment, Biodentine, Cytotoxicity, MTA, Dental pulp stem cells

## Abstract

**Objective:**

The aim of the present study was to evaluate and compare the cytotoxic effects of Biodentine and MTA on dental pulp stem cells (DPSCs) and to assess cell viability and adherence after material exposure to an acidic environment.

**Material and Methods:**

DPSCs were cultured either alone or in contact with either: Biodentine; MTA set for 1 hour; or MTA set for 24 hours. After 4 and 7 days, cell viability was measured using the MTT assay. Biodentine and MTA were also prepared and packed into standardized bovine dentin disks and divided into three groups according to the storage media (n=6/group): freshly mixed materials without storage medium (Group A); materials stored in saline (Group B); materials stored in citric acid buffered at pH 5.4 (Group C). After 24 hours, DPSCs were introduced in the wells and cell adherence, viability, and cellular morphology were observed via confocal microscopy after three days of culture. Cell viability was analyzed using repeated-measures analysis of variance test with Tukey's *post hoc* tests (α=0.05).

**Results:**

Biodentine expressed significantly higher cell viability compared with all other groups after 4 days, with no differences after 7 days. Notably, cell viability was significantly greater in 24-hour set MTA compared with 1-hour set MTA and control groups after 7 days. Material exposure to an acidic environment showed an increase in cell adherence and viability in both groups.

**Conclusions:**

Biodentine induced a significantly accelerated cell proliferation compared with MTA. Setting of these materials in the presence of citric acid enhanced DPSC viability and adherence.

## INTRODUCTION

Mineral trioxide aggregate (MTA) is a calcium silicate-based material and has attracted considerable attention because of its excellent biocompatibility, sealing ability, and antimicrobial properties^20,29^. Although it was initially introduced as a material for repair of root perforations, it is currently used in vital pulp therapy, as a root-end filling material, as an apical plug in apexification procedures, and in regenerative endodontic therapy^12,15,28^. Despite its broad spectrum of clinical indications, MTA comes with certain limitations including long setting time, difficult handling, possibility of crown staining, and high cost^4,20^.

Biodentine (Septodont, Saint Maur des Fosses, France), a new calcium silicate-based restorative cement, was recently introduced for endodontic procedures. This bioceramic material is a fast-setting restorative material recommended as a dentin substitute that can be used in similar applications such as MTA^14^. Materials that are intended for endodontic applications should stimulate repair or be biologically neutral in order to promote healing^6^. If, however, endodontic materials are cytotoxic, they can negatively influence the viability of cells and cause cell death by apoptosis or necrosis^8^. Although there is evidence about the excellent biocompatibility of MTA^5,25,29^, limited information is available about the possible cytotoxicity of Biodentine^2,13,14,17^.

Clinicians often face the challenge of placing materials in a low pH environment due to the presence of inflammation^18^. Variations in the pH at the time of placement could affect the physical and chemical properties of both MTA and Biodentine^5,19,24,31^. It has been shown that the low pH of the surrounding microenvironment affects the hydration reaction of MTA^16^, and that the more acidic the MTA solution during the setting process is, the more extensive its porosity will be^19^. However, limited scientific data exist about the viability and adherence of cells to these materials after the exposure to an acidic environment.

The primary aim of this study was to evaluate the viability of dental pulp stem cells (DPSCs) when in contact with Biodentine in comparison with MTA. The secondary aim was to examine whether the presence of these materials in an acidic environment could have an effect on the DPSCs viability and adherence to these materials.

## MATERIAL AND METHODS

### Cell culture

Human DPSCs were provided by ProCell, Biotechnological Application SA (Athens, Greece). The cells were screened with Flow Cytometry for mesenchymal surface markers. The triple panel of protein CD73, CD90, and CD105, which by consensus is expressed in mesenchymal stem cells, was detected in high levels (>85%). Moreover, the cells were negative for CD45 (hematopoietic cell marker), CD34 (hematopoietic stem cell marker), and CD31 (endothelial cell marker). DPSCs were cultured in basal culture media composed of Dulbecco's Modified Eagle's Medium (DMEM; Gibco, Glasgow, UK) supplemented with 10% fetal bovine, 1X L-glutamine (Gibco), penicillin (100 U/mL; Gibco) and streptomycin (100 mg/mL; Gibco). After reaching 70–80% confluency, cell were treated with 0.05% trypsin (Gibco, Carlsbad, CA, USA) and passed to subsequent culture plates or used in experiments. Cells from third to fifth passage were utilized in this study.

### Cell viability

White ProRoot MTA (Dentsply Tulsa Dental Specialties, Memphis, TN, USA) and Biodentine (Septodont, Saint Maur des Fosses, France) were prepared according to the manufacturer's instructions and placed at the bottom of a 48-well plate (n=5/group). Materials were placed at a 2 mm thickness and fully coverage of the bottom of the well was confirmed for all groups. Immediately after placement, materials were allowed to set for one hour at 37°C in 5% CO_2_ and 100% humidity under sterile conditions and 1×10^4^ DPSCs were introduced in each well in direct contact with the materials. To account for the difference in setting time between the testing materials, a group of 24-hour set ProRoot MTA (MTA24h) was also included (n=5). MTA was allowed to set for 24 hours under the same conditions described above before DPSCs were introduced. All groups were incubated at 37°C in 5% CO_2_ and 100% humidity for 4 and 7 days. Cells without materials served as control. Cell viability was measured using methylthiazolyldiphenyl-tetrazolium bromide (MTT) based cell growth determination kit (Sigma Aldrich, St. Louis, MO) according to the manufacturer's instructions. Relative absorbance (λ=570 nm) for each group was detected in a FlexStation 3 Benchtop Multimode Microplate Reader (Molecular Devices, Sunnyvale, CA, USA).

### Specimen preparation for immunofluorescence

36 dentin disks from anterior bovine teeth were horizontally sectioned into 3-mm slices using an Isomet device (Buehler Ltd., Lake Bluff, IL, USA) and the canal space of each dentin slice was enlarged to 2.6 mm in diameter. Biodentine and MTA were prepared according to the manufacturers' instructions under aseptic conditions and packed into the lumen of dentin disks (N=18/group). Specimens from each material group were further divided into three groups according to the storage media and placed in 24-well plates – Group A: freshly mixed materials without storage; Group B: materials with saline as storage medium; Group C: materials with citric acid buffered at pH 5.4 as storage medium (n=6/group). Each specimen was kept in contact with a saline- or citric acid-soaked piece of gauze for 24 hours in room temperature.

### Immunofluorescence and confocal microscopy

After 24 hours of storage, 2.5×10^4^ DPSCs were introduced in the wells in direct contact with the dentin disks. Groups were cultured for 72 hours at 37°C in a 5% CO_2_ humidified incubator. The cells that adhered to the surface of the samples were fixed in 4% PFA for 20 minutes at room temperature, followed by 0.1 M PBS washing twice for 10 minutes. The samples were then stained using Phalloidin-Rhodamin solution (Molecular Probes, ThermoFisher Scientific, Grand Island, NY, USA) at 37°C following manufacturer's protocol to reveal the cytoskeleton and more specifically the actin filaments of the cells. 4′,6-diamidino-2-phenylindole (DAPI, Vectashield H-1200, Vector Laboratories Inc., CA, USA) was added to stain cell nuclei. Samples were viewed with a microscope with Vectashield's coverslips (VECTOR Laboratories, Peterborough, UK), observed under the confocal microscope Leica TCS SP5 (Leica, Wetzlar, Germany), and processed using Leica software, LAS AF (Leica Microsystems GmbH, Wetzlar, Germany).

### Statistical analysis

Data for the cell viability assay were analyzed using one-way repeated analysis of variance (ANOVA) with Tukey's *post hoc* tests to assess pairwise differences. The level of statistical significance was set at a=0.05. The Brown-Forsythe test was performed to assess equality of group variances prior to performing ANOVA. JMP software (SAS Institute, Cary, NC, USA) and Prism 6 (Graph Pad, La Jolla, CA, USA) were used for data analysis. Mean and standard deviation (mean±SD) were reported for summary statistics.

## RESULTS

**Figure 1:**
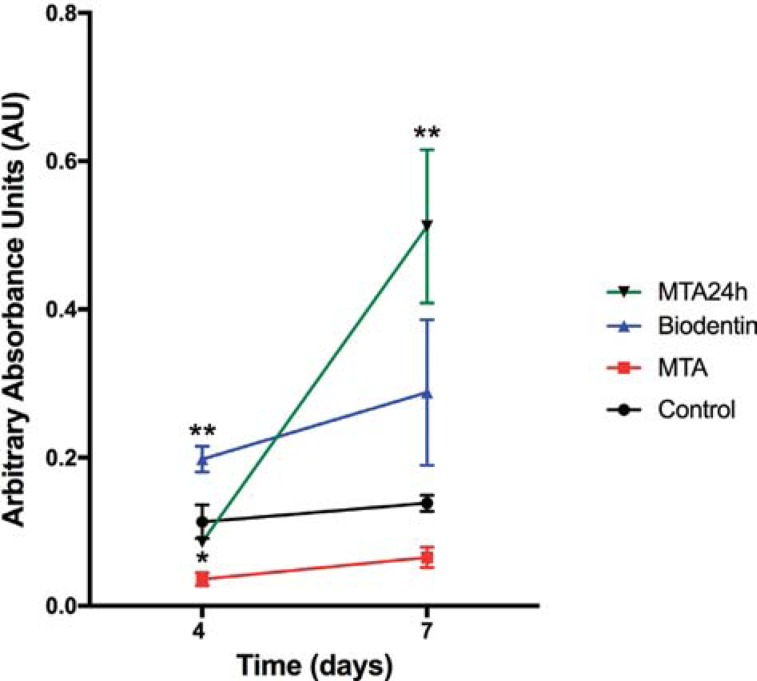
Cell viability as measured by absorbance of human DPSCs after cultured in 24-well plates either alone (control) or in contact with 1-hour set Biodentine (Biodentine), 1-hour set MTA (MTA), or 24-hour set MTA (MTA24h) for 4 and 7 days. Point estimates show mean values for each group and error bars depict standard error. Asterisks denote statistical differences in cell viability between the Biodentine and control group and statistical difference between MTA control and group after 4 days. For the 7-day time point, asterisks denote statistical difference betweenMTA24h and control groups. *P<0.05. **p<0.01

Results from the MTT cell viability assay are shown in Figure 1. Biodentine group expressed significantly higher mean cell viability (0.19±0.03) compared with all other groups after 4 days (all p<0.01). MTA showed significantly lower cell viability (0.03±0.01) after 4 days compared with control group (0.11±0.05) (p=0.01). After 7 days, MTA24h showed significantly higher cell viability (0.51±0.23) compared with MTA (0.06±0.03) (p=0.002) and control group (0.13±0.02) (p=0.009). There was no statistically significant difference between Biodentine and all other groups after 7 days. Intragroup comparisons showed that MTA24h allowed for significant cell viability from 4 to 7 days (p=0.014). A more detailed presentation of the significant intergroup and intragroup pairwise differences is presented in Table 1.

**Table 1:** Summary of the significant intergroup and intragroup pairwise differences in cell viability for the different time points. Mean difference, standard error of mean, and p-value are summarized for each comparison.

Absorbance (4 days)		Mean difference	Standard error of mean	p-value
Biodentine	MTA	0.162	0.021	<0.0001*
Biodentine	MTA24h	0.111	0.021	0.0005*
Biodentine	Control	0.084	0.021	0.0059*
Control	MTA	0.078	0.021	0.0113*
Absorbance (7 days)		Mean difference	Standard error of mean	p-value
MTA24h	MTA	0.447	0.101	0.0023*
MTA24h	Control	0.374	0.101	0.0099*
Absorbance (intragroup differences)		Mean difference	Standard error of mean	p-value
MTA24h (7 days)	MTA24h (4 days)	0.426	0.103	0.0146*

* p<0.05

**Figure 2:**
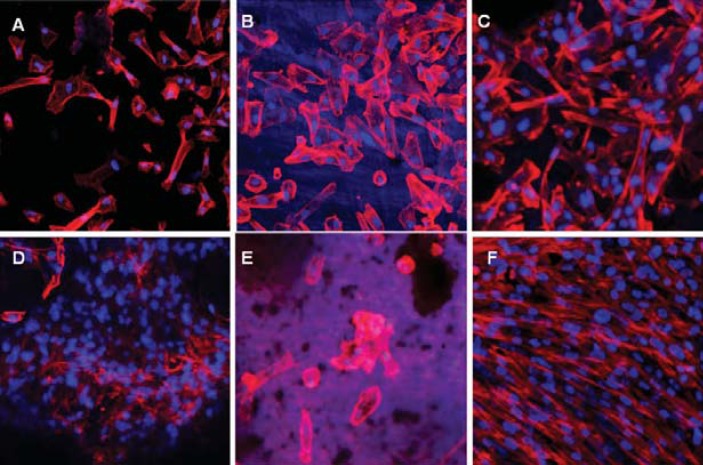
Representative images of the cell cultures on the surface of the different dental materials by confocal microscopy. (A) MTA in no storage media, (B) MTA stored in saline, (C) MTA stored in citric acid, (D) Biodentine in no storage media, (E) Biodentine stored in saline, (F) Biodentine stored in citric acid. Nuclei are identified by DAPI staining (blue). Actin filaments (red) show DPSC adherence and morphology

DPSCs adhered to the surface of dentin disks filled with MTA or Biodentine in all storage media (Figure 2). A higher cell density was observed in the samples of the acidic environment (Figures 2C, 2F) when compared with saline or no storage media. Notably, Biodentine stored in citric acid demonstrated greater amount of adherent cells, thus confirming our results of the cell viability assays. Regarding cell morphology, the cells on materials stored in citric acid demonstrated a typical fibroblast-like appearance with elongated cytoskeleton, whereas cells on materials stored in saline, especially in the Biodentine group, showed similar profiles with milder spindle-shape formation (Figures 2B, 2E).

## DISCUSSION

Materials introduced in procedures, such as vital pulp therapies, regenerative endodontic therapies, or perforation repairs, should primarily possess biocompatibility. MTA is commonly used in such procedures, since it is considered highly biocompatible^25^. Biodentine, a new calcium silicate-based material, has demonstrated biocompatibility when tested on various cell lines with better handling properties and a shorter setting time when compared with MTA^2,13,14,17^. Nevertheless, limited evidence is available regarding Biodentine interactions with dental pulp stem cells^21,33^. This study aimed to investigate the biocompatibility of Biodentine in comparison with MTA on DPSCs in a time course of 4 and 7 days as well as the cell adherence to these materials after exposure to an acidic environment.

Among various advantageous properties of Biodentine is the faster setting time compared with MTA^22^. To compensate for the differences in setting time between the two materials and have comparable results in cell viability, setting-time points of 1 hour and 24 hours were applied for MTA. Results from the cytotoxicity assays after 4 days demonstrated significant DPSC viability when in contact with Biodentine as compared with the other groups. Our data are in consensus with Widbiller, et al.^32^ (2016) who showed that DPSC viability on MTA was significantly lower when compared with that of cells on Biodentine for the first seven days. Nevertheless, other studies have compared the cell viability of human pulp fibroblasts, human gingival fibroblasts, or osteoblast-like cells exposed to Biodentine or MTA and observed similar cell growth without statistically significant differences^2,13,14^. Interestingly, one-hour set MTA was significantly cytotoxic compared with control after 4 days and MTA set for 24 hours demonstrated significantly greater cell viability compared with one-hour set MTA after 7 days. These findings agree with results from a previous study, which showed that apical papilla stem cells expressed significantly greater viability when exposed to 24-hour set MTA as compared with 1-hour set MTA^26^. One possible explanation is that initial release of calcium-ions as well as the presence of leachable and toxic components from fresh MTA may affect the behavior of the cells. It is reported that freshly mixed calcium-silicate based cements may form continuously calcium-silicate hydrates and precipitate calcium-phosphate and calcium carbonate^7^. Nonetheless, MTA and Biodentine had no differences in cell viability after 7 days.

Cell adherence and viability, when in contact with Biodentine compared with MTA, were further assessed using fluorescent probes under confocal microscopy. It has been shown that contact of dental materials with dentin may alter their properties^9^. Thus, the interaction between cement and surrounding dentin was taken into consideration in this study utilizing dentin disks from anterior bovine teeth, since they could be considered an appropriate substitute for human teeth^3^. Confocal micrographs showed that DPSCs attached on the materials stored in citric acid were more sprindle-shaped compared with the materials stored in saline. This fact is indicative of a good cell substrate interaction signifying that both calcium-silicate based materials provide a significantly better substrate for cell adhesion when they set in the presence of citric acid^6,23^. A possible explanation is that the acidic conditions of the citric acid induced the release of Ca-ions, and subsequently the relative concentration of Si increased^30^. Furthermore, the acid-etching effect leads to microstructural changes that could affect the adhesion and proliferation of cells on calcium silicate-based materials^1,10,19,27^. The results of this study agree, despite the differences in methodology, with the results of Kang, et al.^11^ (2013), who reported that MTA mixed with citric acid showed favorable biocompatibility. Importantly, our study shows that Biodentine promoted greater cell adherence and viability compared with MTA. Nevertheless, the mechanisms that are responsible for these effects are not completely understood and further research is required to elucidate them. These data may be applied in future studies to modify the surface of the materials to promote better adhesion and sprouting of cells.

## CONCLUSION

Biodentine demonstrated a significantly faster cell proliferation compared with MTA and control groups. Furthermore, 24-hour set MTA allowed for greater cell viability compared with 1-hour set MTA after 7 days. Both 1-hour and 24-h set MTA were initially more cytotoxic compared with Biodentine, however, there were no significant differences at the 7-day time point. Exposure of MTA and Biodentine to an acidic environment showed an increase in the number of DPSCs adhered to their surface.
